# Increased atherogenic index in the general hearing loss population

**DOI:** 10.1515/med-2020-0003

**Published:** 2020-05-23

**Authors:** Huai Zhang, Dahui Wang, Haiyan Ma, Ying Ren, Chenhui Li, Yihua Zheng, Xiaoming Dai, Lei Yang, Liangwen Xu

**Affiliations:** The Medical School, Hangzhou Normal University, Hangzhou, Zhejiang, China; Breath internal medicine department, The People’s Hospital of Jiangshan, Jiangshan, Zhejiang, China; Hospital director’s office, Tonglu First People’s Hospital, Hangzhou, Zhejiang, China

**Keywords:** atherogenic index, hearing loss, general population

## Abstract

**Purpose:**

The purpose of this study was to evaluate the association of hearing loss with atherogenic index (AI) in the general population.

**Methods:**

A multistage study using cluster random sampling method was conducted in the Zhejiang province from 2016 to 2018. Pure-tone air-conduction hearing thresholds were measured at frequencies of 0.125–8 kHz for each subject. After obtaining their consent, all participants were asked to provide their own plasma lipid data.

**Results:**

A total of 3,414 eligible participants were included, 1,765 (51.7%) were men and 1,649 (48.3%) were women and 1,113 (32.6%) had hearing loss. Ridge regression showed increased AI in subjects with hearing loss. The subgroup with the highest quartile of AI, presenting the highest risk of hearing loss as compared to the lowest quartile, comprised young and middle-aged women. Further analysis revealed that the AI in people with different categories of hearing loss was higher than that in the normal population, except for those with (extremely) severe hearing loss. Moreover, the young and middle-aged women exhibited the most significant correlations between AI and hearing loss.

**Conclusion:**

AI was positively associated with hearing loss in the general population, especially the young and middle-aged women.

## 1 Introduction

As a foremost sensory disorder in humans [1,2], hearing loss leads to communication difficulties, aggravating the unhealthy psychological status of the patients and may, therefore, reduce the quality of life of the patients [3]. Moreover, it may lead to economic losses [4,5]. Furthermore, the 26-year Global Burden of Disease study showed that in terms of both prevalence rate and years lived with disability, hearing loss ranked in the top few places among the 328 diseases and injuries in 195 countries [6].

Atherosclerosis, the main cause of cardiovascular disease, is also one of the major contributors to the global morbidity and mortality [7]. Abundant evidence has confirmed that lipid metabolism disorder is the pathological basis for this disease. Age, infections, inflammation and genetic factors also have been shown to play important roles in atherosclerosis [8,9,10]. Various types of studies including cross-sectional studies [11,12,13], longitudinal studies [14,15,16,17] and intervention studies [18,19] have linked hyperlipidemia to hearing loss. However, these investigations, in general, targeted either specific populations (elderly adults or specific occupational workers) [12,16,17,18] or specific types of hearing loss (presbycusis or sudden sensorineural hearing loss) [11,13]. Additionally, many of these studies included self-reported results for some or all measures (categorical variables) and may lead to the loss of original data and incorporate additional bias [16]. For example, some people did not know they had hyperlipidemia until biochemical tests were done, and these people were likely to fill in the opposite information in the self-reported results.

It has been shown that the ratio of non-high-density lipoprotein cholesterol (non-HDL-C, which is equal to the total cholesterol [TC] minus HDL-C) and HDL-C, known as the atherogenic index (AI) [20,21,22], is a strong predictor of atherosclerosis. The value of AI generally ranges from 0 to 8, with >4.0 to 5.0 (depending on the source) as the generally accepted normal ceiling for a higher risk of cardiovascular diseases [23]. This index has been suggested to be less susceptible to disease activity variation during large intervals, possibly making it more valuable for use in atherosclerosis risk prediction as compared to lipid concentrations. Furthermore, different from single indicators such as TC, HDL-C and low-density lipoprotein cholesterol (LDL-C), just as its formula shows, AI is a comprehensive indicator, simultaneously also with the economical and easy-to-measure characteristics. It contains both non-HDL-C and HDL-C, and the ratio of the two reflects the risk of atherosclerosis, so that an increase or decrease in certain cholesterol (non-HDL-C or HDL-C) does not increase or decrease AI. Moreover, considering this mechanism, i.e., high AI means an increase in intima–media thickness and the presence of artery plaque, and these two may be markers of generalized vascular disease including microvascular disease involving the stria vascularis in the lateral wall of the cochlea [15,24]. Therefore, it is reasonable to study the relationship between AI and hearing loss. Additionally, some studies have reported significant correlation between AI (non-HDL-C/HDL-C ratio), other chronic diseases (such as chronic kidney disease [25] and diabetic heart disease [26]) and exercise [27]. Hence, in the present study, the data from audiometric measurements and routine medical examinations were collected to evaluate the lipid profile and AI in the general population with hearing loss.

## 2 Methods

### 2.1 Study areas and participants

A multistage study using cluster random sampling method was conducted in the Zhejiang Province from September 2016 to November 2018, and the data from six health-care centers were collected and collated. These selected centers were as follows: one in Jiangshan, one in Jiaxing, one in Huzhou and three in Hangzhou (Tonglu county, Baiyang community, and Sijiqing community). A total of 5,303 subjects agreed to provide the relevant information and consented to its use in the future research.

The entire process of the study was approved by the Institutional Review Board of Hangzhou Normal University (grant number 2017LL107). All subjects provided written informed consent, and this research followed the tenets of the Declaration of Helsinki published in 1964 and its later amendments and also the local government policies.

### 2.2 Test procedures

**Figure 1:**
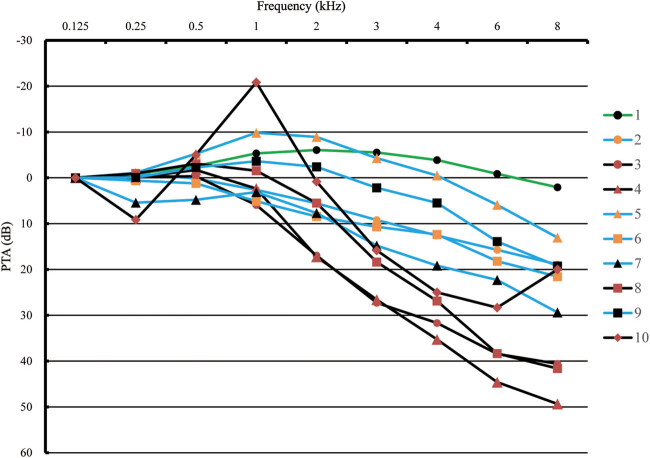
Ten audiogram patterns of hearing loss group classified by *K*-mean cluster analysis. All audiogram shapes are calibrated at 0.125 kHz for facilitating comparison. Hearing loss cases are divided into three subgroups: flat shape (line 1), 2–4 kHz AL shape (lines 3, 4, 8 and 10) and sloping shape (lines 2, 5, 6, 7 and 9).

First, through otoscopy, participants with preexisting ear diseases (such as otitis externa, otitis media or cerumen impaction) or abnormal ear structure were excluded from the analysis (*n* = 111). Furthermore, we excluded participants with missing information on lipid profiles including triglycerides (TGs; *n* = 966), TC (*n* = 1,111), LDL-C (*n* = 1,642) and/or HDL-C (*n* = 1,640; the above excluded numbers are not mutually exclusive), leaving 3,414 participants. All pure-tone air-conduction hearing thresholds were measured by trained researchers using audiometers (AT235; Interacoustics AS, Assens, Denmark) with supra-aural headphones (TDH-39; Telephonic Corporation, Farmingdale, USA). Each participant was evaluated for hearing thresholds between 0.125 and 8 kHz (0.125, 0.25, 0.5, 1, 2, 3, 4, 6 and 8 kHz) over an intensity range of −10 to 110 dB in a soundproof booth with a background noise of less than 20 dB(A). All facilities were calibrated before use, and we conducted the procedure by beginning at 1 kHz, continuing to higher test frequencies and then returning to 1 kHz, followed by testing lower frequencies, and the same test method has been described in detail in previous study [28]. Presently, hearing loss was defined as the pure-tone average (PTA) of the speech frequencies (0.5, 1, 2 and 4 kHz) of ≥26 dB in the better ear [4]. Additionally, the degree of hearing loss was classified into one of the four categories according to PTA (mild, 26–40 dB; moderate, 41–60 dB; severe, 61–80 dB; and extremely severe, ≥81 dB). Furthermore, based on the nine-frequency hearing threshold data from the better ear, each participant had his or her own audiogram shape. Therefore, the audiogram shapes of all patients with hearing loss were clustered into ten categories for an in-depth analysis of the relationship between hearing loss and AI. According to the AMCLASS™ standard description [29] and the application of the protocol described by Liu et al. [30], the audiogram shapes could be described as follows based on the *K*-mean cluster analysis: a flat shape, a 2–4 kHz abrupt loss (AL) shape and a sloping shape (Figure 1). A flat shape is the audiogram where all the octave thresholds fluctuate within ±10 dB difference to the mean (line 1), a 2–4 kHz AL shape is where a difference of more than 15 dB exists between 2 and 4 kHz (lines 3, 4, 8 and 10), and a sloping shape is a general downward trend of any slope with differences not more than 10 dB between the adjacent frequencies (lines 2, 5, 6, 7 and 9).

Moreover, the blood samples were drawn from the participants in the morning (the same day as the audiometric testing) following an overnight fast and measured at the clinical laboratories of each health-care center. Due to inconsistencies in the items examined by each center, some plasma lipid indicators (such as apolipoprotein A1, apolipoprotein B and lipoprotein A) were eventually not included in the analysis.

### 2.3 Statistical analyses

All raw data were documented and verified (double entry and validation) via EpiData 3.1 (The EpiData Association, Odense, Denmark). The statistical package for the social sciences (SPSS) software (version 19.0 for Windows; SPSS Inc., Chicago, USA) was used to conduct all statistical analyses, and the results were graphically represented using the Adobe Illustrator CS5 software (Adobe Systems Inc., San Jose, USA). Data were presented as mean ± standard deviation or median (interquartile range) according to the distribution of each variable as examined by the Kolmogorov–Smirnov normality test. Student’s *t* test, *χ* ^2^ test and Wilcoxon rank sum test were used to compare the differences between the groups, and the Bonferroni correction was applied for multiple comparison. Multicollinearity was assessed by computing the variance inflation factors (VIFs) to determine whether multiple linear regression or ridge regression analysis [31,32] is used to estimate the correlation between the PTA and the variables. Figure S1 shows the independent variables to be highly correlated (VIF > 10). Herein, the ridge regression, a regularization method dedicated to collinear data analysis, was a very suitable statistical method to replace multiple linear regression without losing data. Furthermore, it performs well with multiple predictors, each having small effect, and prevents the coefficients of linear regression models (with multiple correlated variables) from being poorly determined and exhibiting high variance [32]. Additionally, the *K*-mean cluster analysis method (a gold-free standard [unsupervised learning] classification method that allows multiple explorations of the same problem) was used to classify the patients’ audiogram shapes into several categories for analysis. Furthermore, the analysis of variance test and logistic regression were conducted to analyze the hearing threshold differences between different hearing loss levels and audiogram shapes, after adjusting (and/or stratification) for age and sex. To assess the consistency of the findings across participant characteristics, we estimated the odds ratio (OR) and 95% confidence interval (CI) of hearing loss for a doubling in AI concentration [33,34]. All the reported probability values were two tailed, and the statistical significance was determined at the 0.1 level for the normality test and the 0.05 level for all the other effects.

## 3 Results

### 3.1 Demographic and clinical characteristics

Of the 3,414 eligible participants, 1,765 (51.7%) were men and 1,649 (48.3%) were women, and a total of 1,113 (32.6%) had hearing loss. Table 1 shows the results of the comparison between the demographic characteristics and plasma lipid profiles of the participants. Compared with the normal subjects, the patients with hearing loss were older (62.83 ± 11.49 years versus 47.45 ± 13.40 years), and males constituted a larger proportion of those affected (56.1% versus 49.6%). The TG ( *p* = 0.041), TC ( *p* < 0.001), LDL-C ( *p* < 0.001) levels and AI ( *p* < 0.001) were significantly higher, whereas the HDL-C level ( *p* = 0.022) was significantly lower in the hearing loss group.

**Table 1:** Comparison of demographic and clinical characteristics between hearing loss and the normal group

	Total	Speech-frequency hearing loss		*p*
		No	Yes	
Number	3,414	2,301	1,113	
Age (years)	52.46 ± 14.70	47.45 ± 13.40	62.83 ± 11.49	<0.001
Sex (men/women)	1,765/1,649 (51.7%/48.3%)	1,141/1,160 (49.6%/50.4%)	624/489 (56.1%/43.9%)	<0.001
TG (mmol/L)	1.30 (0.91, 1.90)	1.29 (0.90, 1.88)	1.33 (0.94, 1.96)	0.041
TC (mmol/L)	4.95 ± 0.94	4.91 ± 0.92	5.04 ± 0.99	<0.001
LDL-C (mmol/L)	2.87 ± 0.81	2.83 ± 0.78	2.95 ± 0.86	<0.001
HDL-C (mmol/L)	1.43 ± 0.34	1.44 ± 0.35	1.41 ± 0.34	0.022
AI	2.62 ± 0.94	2.56 ± 0.91	2.73 ± 0.99	<0.001

Note: *p* values based on Student’s *t* test for continuous variables, *χ* ^2^ test for categorical variable (sex) and Wilcoxon rank sum test for abnormal distribution variable (TG). Data were presented as proportions, M ± SD or median (interquartile range) according to the original data distribution.

Abbreviations: TG, triglyceride; TC, total cholesterol; LDL-C, low-density lipoprotein cholesterol; HDL-C, high-density lipoprotein cholesterol; AI, atherogenic index.

### 3.2 Correlation between plasma lipid profiles and PTA

Since the relevant plasma lipid indexes and the hearing thresholds were collected as quantitative data, and a high correlation existing among the independent variables (Figure S1), ridge regression is considered an appropriate method to analyze the relationship between PTA and plasma lipid indexes. In addition to age and sex as the indicators of lipid parameters, AI showed significant positive correlation with PTA (*B* = 0.20, *p* = 0.007; Table S1). Furthermore, when stratified by sex and age (60 years being the demarcation point between the elderly and the young and middle aged; Table 2), the subgroup with the highest quartile of AI, i.e., the young and middle-aged women (OR = 2.81, 95% CI: 1.76–4.47, *p* < 0.001), presented the highest risk of hearing loss when compared to those with the lowest quartile.

**Table 2:** OR (95% CI) of hearing loss by AI levels stratified by sex and age

	*N*	For men		*N*	For women	
		OR (95% CI)	*p*		OR (95% CI)	*p*
AI quartile						
For age ≤60 years						
Q1 (<1.929)	183	1 (reference)		418	1 (reference)	
Q2 (1.929–2.502)	271	1.42 (0.88–2.30)	0.149	326	1.43 (0.94–2.18)	0.096
Q3 (2.503–3.155)	335	1.34 (0.84–2.14)	0.216	226	1.72 (1.10–2.69)	0.018
Q4 (>3.156)	385	1.35 (0.85–2.13)	0.200	151	2.81 (1.76–4.47)	<0.001
For age >60 years						
Q1 (<1.929)	144	1 (reference)		100	1 (reference)	
Q2 (1.929–2.502)	142	0.87 (0.53–1.41)	0.559	128	0.53 (0.31–0.91)	0.022
Q3 (2.503–3.155)	153	0.59 (0.37–0.95)	0.028	142	0.56 (0.33–0.95)	0.032
Q4 (>3.156)	153	1.06 (0.65–1.73)	0.815	157	0.90 (0.53–1.53)	0.706

### 3.3 Relationship between AI and the different types of hearing loss

According to Section 2, the audiogram shapes of all hearing loss patients were classified into three categories in this study, such as a flat shape (*n* = 191), a 2–4 kHz AL shape (*n* = 256), and a sloping shape (*n* = 666). Table S2 presents the estimated changes in risk associated with AI levels in different hearing groups through different adjusted covariates. Without adjustment for age and sex, the per doubling increase in AI was significantly associated with a 62%, 50% and 32% increase in the risk of hearing loss (for flat shape, OR = 1.62, 95% CI: 1.21–2.17; for 2–4 kHz AL shape, OR = 1.50, 95% CI: 1.19–1.94; for sloping shape, OR = 1.32, 95% CI: 1.11–1.56), respectively (Table S2). Furthermore, based on an in-depth analysis, after stratifying by sex and age (Table 3), increased AI was found to be associated with incidences of hearing loss in young and middle-aged women (for 2–4 kHz AL shape, OR = 2.19, 95% CI: 1.52–3.17, *p* < 0.001; for sloping shape, OR = 1.44, 95% CI: 1.16–1.80, *p* = 0.001). However, the OR between the flat shape subgroup and the normal group failed to achieve statistical significance.

**Table 3:** Association between AI and the audiogram shapes of hearing loss stratified by sex and age

Audiogram shapes	For men				For women			
	*N*	AI	OR (95% CI)	*P*	*N*	AI	OR (95% CI)	*P*
For age ≤60 years								
Normal	927	2.86 ± 0.97	1 (reference)		937	2.24 ± 0.79	1 (reference)	
Flat shape	74	2.97 ± 1.14	1.12 (0.89–1.42)	0.346	35	2.45 ± 0.81	1.39 (0.94–2.05)	0.102
2–4 kHz abrupt loss	52	2.85 ± 0.91	0.99 (0.74–1.33)	0.947	26	2.91 ± 0.99^a^	2.19 (1.52–3.17)	<0.001
Sloping shape	121	2.95 ± 0.87	1.10 (0.91–1.33)	0.346	123	2.48 ± 0.82	1.44 (1.16–1.80)	0.001
For age >60 years								
Normal	215	2.59 ± 0.85	1 (reference)		222	2.67 ± 0.76	1 (reference)	
Flat shape	35	2.98 ± 0.96	1.45 (1.04–2.03)	0.028	47	2.65 ± 0.96	0.98 (0.69–1.39)	0.901
2–4 kHz abrupt loss	120	2.73 ± 1.03	1.17 (0.93–1.47)	0.192	58	2.64 ± 0.93	0.96 (0.69–1.33)	0.812
Sloping shape	222	2.60 ± 1.02	1.01 (0.83–1.24)	0.915	200	2.81 ± 1.05	1.18 (0.96–1.45)	0.121

^a^The index is the largest with significant difference in the subgroup.

**Figure 2:**
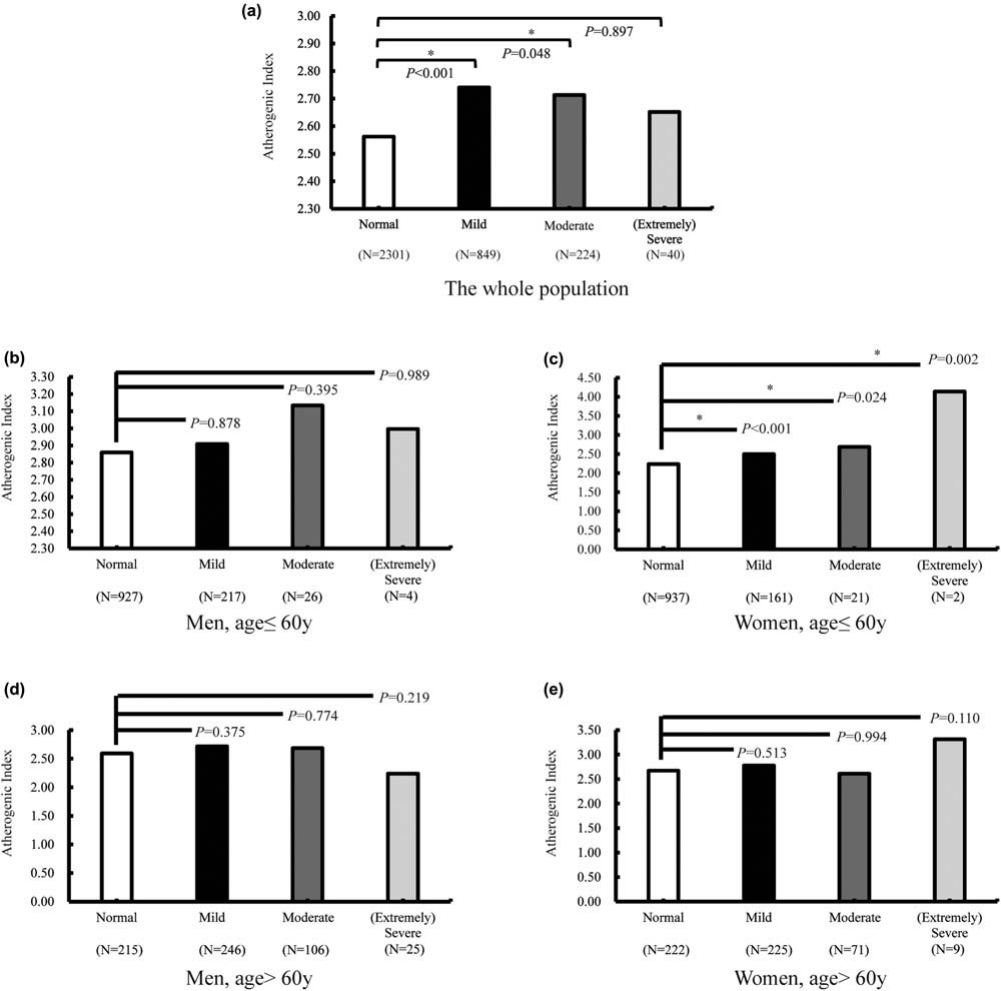
AI of different subgroups (a: the whole population; b: men, age ≤60 years; c: women, age ≤60 years; d: men, age >60 years and e: women, age >60 years). The values corresponding to the ordinate unit length of each subfigure are different.

As described in Section 2 of this study, according to the standards recommended by the World Health Organization in 1997 [35], the hearing levels were classified into four categories such as normal (*n* = 2,301), mild (*n* = 849), moderate (*n* = 224) and (extremely) severe (*n* = 40; the severe and extremely severe population were merged due to the small number in both the subgroups). As shown in Figure 2a, after adjusting for age and sex, the AI in people with mild hearing loss was significantly higher than that in the normal population (*p* < 0.001). Additionally, with progressive increase in the degree of hearing loss, the AI gradually decreased, and no significant difference was observed between the (extremely) severe subgroup and the normal group (*p* = 0.897). The results, after stratification for sex and age (Figure 2b–e), showed that the AI value for each degree of hearing loss was significantly higher in the young and middle-aged women as compared to the normal population. Notably, although the number of (extremely) severe subgroup was too small to be meaningfully compared with other subgroups, the AI among other types of hearing population still had a comparative significance.

## 4 Discussion

This cross-sectional study, conducted in a large population and based on a cohort of local individuals in the Zhejiang province, provides information about AI in subjects with hearing loss in general population. The age, sex and lipid-related biochemical markers (especially AI) of the hearing loss population are the contents of this paper. Through appropriate statistical analysis methods, the relationship between AI and different hearing losses in different groups is discussed in detail. It is hoped that some research results in this study could provide new ideas or directions for future study on hearing loss.

Different from other studies [11,16], the present study collected original quantitative data for each participant’s plasma lipids as well as their continuous hearing data. Particularly, we introduced a comprehensive indicator, AI, an economic, easy-to-measure, and noninvasive lipid parameter, derived from certain lipid markers. Based on the standard definition (i.e., the average of 0.5, 1, 2 and 4 kHz hearing thresholds of ≥26 dB in the better ear), 32.6% of the participants were found to have hearing loss. Consistent with other studies [36,37], we found age and sex to be closely related to hearing loss. Specifically, the prevalence of hearing loss was higher in males and the elderly population.

Due to the existence of multicollinearity among the independent variables, the precondition for applying multiple linear regression was not met. Herein, the ridge regression analysis, a specialized processing statistical analysis that deals with multicollinearity problem, is a suitable alternative to assess the correlation. Ridge regression results showed that the relationship between single plasma lipid indicator and hearing loss was not significant; however, the comprehensive indicator, AI, significantly correlated with hearing loss. The above results were partially consistent with previous studies [14,15], wherein all indicated no significant correlation between single plasma lipid indicator (non-HDL-C in previous studies) and hearing loss. One difference between this research and previous studies was the difference in the criteria for determining hearing loss (the better ear versus the other ear). The model suggested that non-HDL-C levels increased in patients with hearing loss, while their HDL-C decreased, when compared with the normal population. Simultaneously, it is worth noting that AI is a strong indicator for predicting the risk of cardiovascular diseases (including atherosclerosis). Remarkably, although in the current study, the average of AI in both the normal and hearing loss group was far less than 4, statistically significant differences were observed between the two groups, and AI was observed to be higher in patients with hearing loss. Insufficient blood supply to the cochlea may be one of the reasons for hearing loss; since the cochlea is very vulnerable to ischemic injury and lacks collateral circulation besides the cochlear artery (supplied by the labyrinthine artery) [11,38]. High non-HDL-C and low HDL-C levels may lead to endothelial damage in the peripheral arteries, thereby leading to hearing loss due to circulation disturbances at the cochlear end of the artery [39,40]. On the contrary, numerous experimental studies have shown that an abnormal increase in reactive oxygen species has a significant damaging effect on the ear cells [41,42,43]. Hence, an increase in the level of oxidative stress induced by high fat (possibly leading to *in vivo* imbalance of oxidation/antioxidation [44,45]) may be another explanation for hearing loss in high AI subjects.

The patients with hearing loss were grouped according to the different criteria for further analyses. The differences in audiogram shapes of people with hearing loss introduce no change in the results, since the overall AI values in the hearing loss group were higher than that in the normal group. Additionally, compared with the normal group, after adjusting for age and sex, AI was significantly different in the mild hearing loss subgroup but not in the (extremely) severe subgroup (Figure 2a). The results of mild hearing loss and moderate hearing loss were consistent with the result of the ridge regression. Nevertheless, further research, especially follow-up studies, are needed in support of this hypothesis. The observed negative result may be due to the small sample size of the (extremely) severe subgroup (*n* = 40).

On the contrary, in addition to adjusting for sex and age, our study found that young and middle-aged women were more sensitive to AI changes through stratified analysis (Tables 2, 3 and Figure 2). This result was not inconsistent with the study conducted by Simpson et al., after all their research object was only the elderly. Moreover, analysis of the elderly population in this study also reached similar conclusion that the association was either spurious or too small to be of consequence in the assessment and treatment of hearing loss in older adults [17]. Besides, it is worth noting that the number in some subgroups is much smaller than the normal population, especially the number in the (extremely) severe hearing loss subgroup (*n* = 40), and subsequently, poststratification, the number of participants will be even smaller.

The limitations of this study need to be noted. First, a causal relationship could not be established due to the cross-sectional study nature. Second, this study did not exclude people with hearing loss caused by ototoxic drugs or noise trauma, as these data were not collected. Third, plasma lipid data from different regions were measured in the clinical laboratories of the respective health-care centers, therefore, the possibility of detection bias cannot be excluded. Fourth, because the parameters including lifestyle factors (such as exercise and diet), genetic factors, systolic and diastolic blood pressure, apolipoprotein A1 and other physiological and biochemical indicators were missing or not examined, the aforementioned factors could not be included in the analyses.

## 5 Conclusion

In this population-based cross-sectional study which analyzed various variables and quantitative data, we found AI to be positively associated with hearing loss in the general population, especially, the young and middle-aged women. These results may provide clues to delineate the mechanism of hearing loss. Nevertheless, possibly the most critical for public health is that elevated AI can elicit adverse effects on hearing even at levels well below the critical value (<4), which increase the importance of maintaining the results that suggest low level of AI.
